# Is technological-assisted leg axis alignment reliable in total knee arthroplasty with varus–valgus deformities?

**DOI:** 10.1007/s00590-025-04551-7

**Published:** 2025-10-07

**Authors:** Daniel Hernandez-Vaquero, Alfonso Noriega-Fernandez, Sergio Roncero-Gonzalez

**Affiliations:** University of Oviedo, Oviedo, Spain

**Keywords:** Total knee arthroplasty, Varus-valgus deformities, Alignment, Digital x-ray, Computer-assisted system, Technology-assisted TKA

## Abstract

**Purpose:**

Computer-assisted systems improve conventional instrumentation in normalizing the axis of the limb in frontal deformities. The performance of a postoperative X-ray has been questioned to verify this axis, assuming that it can be replaced by the final image that appears on the system monitor. The objective of this work is to verify whether both axes are similar.

**Methods:**

In 123 patients with varus–valgus frontal deformity, a total knee arthroplasty was implanted with the aid of the same navigation system without previous images. On the one hand, the final femorotibial angulation that appeared on the monitor screen, and on the other hand, the one observed in a long postoperative digitalized radiograph were recorded.

**Results:**

A discrepancy and poor correlation were observed between the femorotibial alignment verified radiographically and that shown on the system monitor at the end of the surgical procedure. Differences were significant in the valgus deformity group (*p* = 0.04) and especially in the varus cases (*p* < 0.001). The median postoperative axis as measured by computer-assisted was very close to neutral, but a deviation toward varus was observed on the X-ray.

**Conclusion:**

The axis of the limb observed with a computer-assisted system is closer to neutral alignment than that shown on the digitalized X-ray image after total arthroplasty in patients with frontal deformities. A postoperative X-ray is recommended as certification of the result, being more reliable than the final image from the system monitor.

## Introduction

Despite the excellent results provided by total knee arthroplasty (TKA) [1], in a significant percentage of cases, the expected functional benefit is not achieved. Numerous factors are known that may explain these defective results [2]. It is possible that the patient has higher expectations about the result of this procedure and expects more in terms of functionality of the knee and return to normal life. An extensive literature has investigated the cause of these poor results, revealing multiple reasons, and among them, abnormal postoperative alignment of the implant has been discussed [3].

The good alignment of the TKA following the normal axis of the limb has been one of the most pursued purposes in this procedure. Traditionally, it has been accepted that a neutral mechanical axis of the limb or in slight valgus is the ideal goal after TKA implantation [4].

The use of computer technology, technologically assisted systems (TAS), allows for accurate preoperative planning and intraoperative verification of the position, alignment, size, and function of the arthroplasty. It can be considered a remarkable aid in achieving proper limb alignment and ligament balance. A high number of TKAs are implanted with TAS, and is considered a valid and well-documented option [5], just as superior long-term clinical results have not been demonstrated compared to the conventional technique in cases without deformity [6], TAS has shown its usefulness in obtaining correct alignment in cases with varus or valgus deformities [7]. Patients with these pre-existing deformities represent a challenge for the surgeon and achieving a neutral axis is a strongly sought goal [8].

The femorotibial alignment obtained at the end of the procedure is shown on the monitor screen when using TAS and on the postoperative X-ray that includes the entire leg. The possibility of knowing this axis by TAS has led to the assumption that it would not be necessary to perform postoperative radiographic control, accepting as the final mechanical axis of the limb the one collected in the last stage of the surgical procedure. The main objective of our study is to find out if there are differences in the final correction obtained after TKA, analyzing the radiographic measurement and the TAS monitor. The secondary objective is to find out if any factor can alter the relationship between both techniques. In summary, the aim is to find out whether in cases with previous frontal deformity, the leg alignment shown on the final screen of a TAS is like that shown in the postoperative radiographic control.

## Methods

### Patient selection

This was a cross-sectional analytical observational prospective, non-randomized study. Specific informed consent was obtained from all the participants included in the study. This work was approved by the Regional Ethics Committee (PI12/01098). The series consists of 123 cases of patients in whom the same TKA model was implanted with the aid of the same navigation system and in whom a varus or valgus deformity greater than 3° was observed radiographically. The cases were selected after performing long radiographs including hip, knee and ankle (PreRx) in which a metallic reference of known diameter was included in the preoperative study. Using a computer program (Impax 6.3.1.2813, Agfa Healthcare N.U. Montsel, Belgium), the images were sent to the surgical planning software (Agfa Orthopaedics Tools *v* 2.06). This tool was used to calculate the anatomical and mechanical axes of the femur and tibia and then the anatomical and mechanical axes of the leg [9]. Varus angulation was considered positive, and valgus angulation was negative. Deformity was defined as varus when the femorotibial mechanical axis was ≥ 183° and valgus when the femorotibial mechanical axis was ≤ 177°. Measurements were performed by two of the authors, who had extensive experience in the use of this planning system.

### Surgical technique

All patients were operated on by the same surgical team, and in all patients, the same prosthetic model was implanted. Also in all cases, an identical closed navigation system without prior imaging (OMNIBotics system, Corin Group, Cirencester, UK) was used. After removal of the osteophytes, a tibial cut was first made at 90° on the mechanical axis of the tibia in the coronal plane with 5° posterior slope in the sagittal plane; the initial femorotibial gap was recorded in extension. Under vision on the navigator monitor, the femoral cut was then performed applying the dependent cutting technique. By sequential releases with the aid of a distractor, symmetrical spaces were obtained in extension and flexion with equal soft tissue tension until a neutral mechanical axis of 180 ± 3° was achieved. After placement of the TKA and before the end of the procedure, the femorotibial alignment achieved (PosNv) shown on the last screen was recorded and filed in the personal computer file of each case. All patients underwent a long-term radiograph (PosRx) 30 days after TKA, using the same technique as mentioned in the preoperative study.

### Statistical analysis

Quantitative variables were expressed as mean ± SD if followed a normal distribution or median (p25–p75) if not. Categorical variables were expressed as *n*(%). Comparisons between numerical variables were studied with Student’s t-test and for categorical variables with the Chi-square test. Pearson’s test was also used to find the correlation coefficient between the PosRx and PosNv variables. 0.05 was considered the level of statistical significance. A multiple linear regression was performed to determine to what extent the difference between PosRx and PosNv was influenced by preoperative parameters: radiographic alignment, age, sex and BMI. Statistical program Stata, v16 (StataCorp LLC, Texas, USA) was used.

## Results

### Demographics

Of the 123 patients, 38 had a preoperative valgus axis and 85 had a varus axis. The median of the patients in valgus was 169.1° and in varus 191.9°. The medium mechanical femorotibial axis of the entire series according to the preoperative radiograph (PreRx) was 187.9° (174.3–193.9). The mean age was 71.4 years (65–79. There was no difference in age between valgus and varus cases. The median BMI of the series was 31.1 (27.1–34.4), with no statistical differences between the varus and valgus groups (*p* = 0.09). The female sex predominated in the series, being more frequent in valgus cases with a significant difference (*p* = 0.003) (Table 1).

**Table 1:** Epidemiological characteristics of the series

	General series (*N* = 123)	Valgus group (*N* = 38)	Varus group (*N* = 85)	*P* values
BMI	31.7	29.1	31.4	0.09
Mean age	71.4	70.1	72	0.31
Female gender (%)	65.4	84.2	56.5	0.003

### Measurements of the complete series

A discrepancy was observed between the median postoperative alignment according to the monitor screen, where all patients had an alignment of 180 ± 3°, and that measured on radiography where three cases ended up with valgus deformity and 23 had some varus deformity. The median postoperative axis according to the navigation monitor display (PosNv) was 180° (180–181). The postoperative radiograph (PosRx) showed a greater deviation toward the varus reaching 182.1° (180.6–184.1) (*p* < 0.001) (Table 2).

**Table 2:** Median values of postoperative angulation navigator screen (PosNv) and radiograph (PosRx))

	PosNv	PosRx	*P* values
General series	180° (180–181)	182.1° (180.6–184.1)	< 0.001
Valgus group	180° (180–182)	180°.8 (179.5–182.1)	0.04
Varus group	180° (179–180)	183° (181.5–184.3)	< 0.001

### Valgus and varus group measurements

In the valgus group, the median PosRx angulation was 180.8° (179.5–182.1), while the PosNv angulation was 180° (180–182). In the varus group, the median PosRx was 183° (181.5–184.3), while the PosNv was 180° (179–180). The difference between the angulation obtained in the PosNv and PosRx measurement in valgus patients was significant (*p* = 0.04) and highly significant in varus (*p* < 0.001). A greater correction of valgus cases than varus cases was observed in both PosRx and PosNv (Table 3) considering the correct axis when it is located between 177° and 183°. Of 85 patients that started with varus, 23 ended up with some varus deformity in the PosRx measurement and only four in the PosNv measurement. Of 38 patients in valgus, three ended up with some valgus deviation in the PosRx measurement and none in the PosNv.

**Table 3:** Correction of preoperative deformity at the end of the surgical procedure

Axis correction	Varus group (*N* = 85)	Valgus group (*N* = 38)	*p* values
According to the X-ray	62	35	0.017
Based on monitor TAS	81	38	0.31

**Fig. 1:**
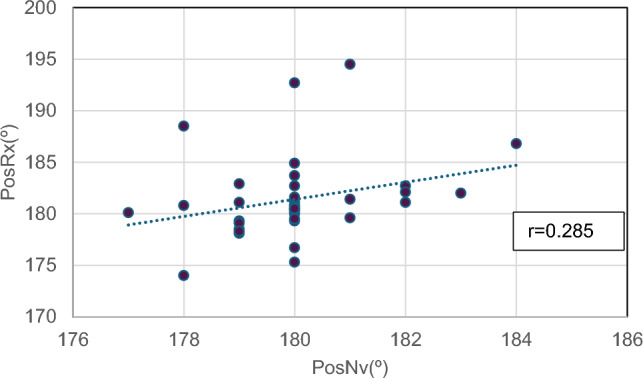
Correlation between radiographic alignment and that shown on the monitor screen in the valgus group. *r* = Pearson’s correlation coefficient

**Fig. 2:**
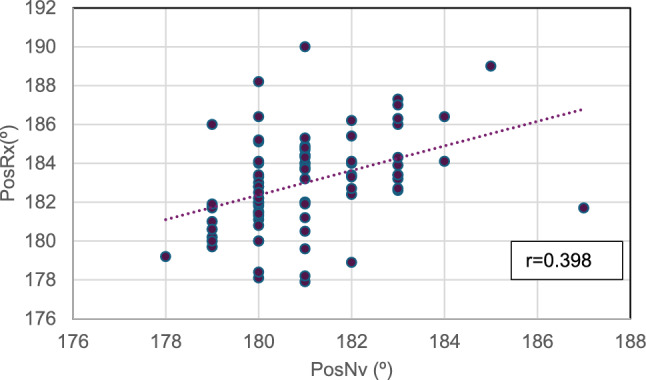
Correlation between radiographic alignment and that displayed on the monitor screen in the varus group. *r* = Pearson’s correlation coefficient

An independent multiple linear regression was performed in both varus and valgus groups including age, BMI, sex and preoperative radiographic alignment (PreRx) as influencing factors. In the valgus group, no effect of any variable was observed, but in the varus group, there was a linear relationship between the severity of the preoperative alignment and the male sex, which could be affected in such a way that for each degree that the preoperative alignment increased, the difference between PosRx and PosNv increased by 0.1°. The male sex showed a difference between PosRx and PosNv of 0.8° (Table 4). Pearson’s correlation coefficient between PosNv and PosRX was 0.237 in general series (*p* = 0.152), 0.285 (*p* < 0.08) in valgus patients (Fig. 1) and 0.398 (*p* < 0.001) in varus (Fig. 2).

**Table 4:** Multiple linear regression in the varus group

Dif	Coef	Std.err	*t*	*P* > *t*	95% Conf.interval
PreRx	.1169566	.0315784	3.70	0.000	.0541014 .1798118
Age	.029577	.0214733	1.38	0.172	−.0131645 .0723185
BMC	−0167207	.0230295	−0.73	0.470	−.0625599 .0291184
Sex (M)	.8058632	.361436	2.23	0.029	.0864429 1.525284
Constant	−21.91523	5.9337	−3.69	0.000	−33.72597 −10.1045

## Discussion

The main finding of this work is that after TKA in patients with frontal deformity we observed a discordance and poor correlation between the femorotibial alignment measured radiographically and that shown by the TAS at the end of the surgical procedure. In cases with previous valgus deformity, we found a significant difference between both measurements, a difference that was very significant in varus cases. In addition, the radiographic measurement showed a greater number of cases with a successful correction with valgus deformity than those with varus deformity, which was not the case in the measurement observed on the TAS screen.

Mechanical instrumentation in TKA, which has been developed and improved over time, is not free of errors that can reach 40% of cases [10]. This has encouraged the search for other techniques that would at the same time avoid cruel gestures such as invasion of the spinal canal. Computer-aided TKA implantation allows the creation of a three-dimensional model from the patient’s specific anatomy [11, 12]. Cases with frontal extra-articular deformities have been one of the main indications for TKAs [13], and although there are discrepancies regarding long-term clinical outcomes, this technique has been positively associated with better alignment of prosthetic components, is cost-effectiveness [14] reduces the frequency of medium-term revisions surgery [15].

There are discrepancies in the literature regarding the agreement between the alignment obtained on radiography and that shown by TAS [16, 17]. Systems without prior imaging require detailed intraoperative registration of the articular surfaces and certain bony landmarks to create the surgical model, which is dependent on a manual technique and therefore subject to errors. This may be more prominent in frontal varus–valgus deformities where the bony structures collected may be imprecise or difficult to identify.

In our work, the median postoperative axis of the entire series as measured by TAS was very close to neutral, but nevertheless a deviation toward varus of almost 3° was observed in the radiographic findings. Radiographic correction was not achieved in 27% of cases in varus and in 4.7% in TAS. This high percentage of cases in which radiographic correction was not achieved is striking. In contrast, in the valgus group, radiographic correction was not achieved in 7.9% of cases, but all cases were corrected according to the TAS. Almost one-third of the cases starting from varus ended up with some varus deformity on postoperative radiography, a much higher number than in the TAS monitor. In contrast, valgus cases generally ended up in a neutral axis on both the radiograph and the monitor. Based on our findings, it appears that the TAS monitor findings are more lenient than the radiographic study especially in varus cases. This deviation of the radiographic axis in varus is an original finding of our work and although there is no uniformity in the literature on the clinical consequence of this alteration [18, 19]; it has been related to a lower implant survival [20, 21]. It can be considered, according to our study, that the limb axis observed with a TAS monitor is closer to neutral alignment than the axis shown in the radiographic image.

Some authors [22, 23] also found no concordance between the two measurements, especially in cases of varus deformity, without finding any influential factor. On the contrary, in our study, we observed that male gender and the severity of the previous deformity had an influence in the varus group of patients. Yaffe et al. [24] found a much greater discrepancy than in our work, reaching 8°. This discordance is also noted by other authors who relate it to the support of the limb when taking the X-ray [25] or to the time elapsed since the arthroplasty [26]. Although we do not know the cause of this discrepancy, one cause may be the defective collection and/or registration of the bony references from which the computer system projects the preoperative model [27]. Technology helps to design a three-dimensional model of the leg, but the collection of references and mapping of structures is manual and therefore subject to errors. The recommendations made by the TAS to guide the surgical technique may also be wrong and the resulting axis will be incorrect.

### Limitations

Patients have not been blindly selected. Postoperative radiographic measurement was performed after a short period of time after TKA and was not repeated later. Other radiological techniques such as computed tomography were not used. A single TAS model has been used, and we do not know if other systems would have given different results. An inter- and intraobserver study of the measurements has not been performed. The TAS and the radiographic measurement system already provide the obtained angulation.

## Conclusions

Our results cast doubt on the reliability of the final limb alignment obtained by computer-aided systems based on the design of an algorithm created by manual landmarking. Although the difference in the axis of the limb between the radiograph and the final screen of the TAS is not very wide, its lack of concordance is striking when considering a technique that has precisely this as one of its objectives. The discordance we have found advises against checking postoperative alignment after TKA exclusively with computer techniques.

## Data Availability

No datasets were generated or analysed during the current study.
